# Immunofluorescence staining profiles of glomerular diseases: a single-center retrospective study

**DOI:** 10.1016/j.bbrep.2025.102362

**Published:** 2025-11-14

**Authors:** Jiarong Song, Xinyuan Cui, Shuguang Yuan, Hong Liu, Yu Liu, Xuan Zhou, Lin Sun, Xuejing Zhu, Yifu Li

**Affiliations:** a Department of Nephrology, The Second Xiangya Hospital, Central South University, 139 Renmin Road, Changsha, Hunan, 410011, China; b Center for Medical Research, The Second Xiangya Hospital, Central South University, 139 Renmin Road, Changsha, Hunan, 410011, China; c Department of Nephrology, Affiliated Hospital of Guangdong Medical University, No 57 South Renmin Avenue, Zhanjiang, Guangdong, 524001, China

**Keywords:** Renal biopsy, IgA-associated renal disease, Immunofluorescence (IF), Retrospective study, IgA nephropathy (IgAN), Glomerular disease

## Abstract

Immunofluorescence (IF) staining is essential for diagnosing glomerular diseases, yet comprehensive characterization of immune deposition patterns remains limited. We retrospectively analyzed 10,489 consecutive native kidney biopsies (2011–2022) from a tertiary center. IF staining for IgA, IgG, IgM, C1q, C3, C4d, and fibronectin was semi-quantitatively graded (0–4+) and deposits classified as mesangial area only (MAO), glomerular capillary loop only (GCLO), or combined mesangial–capillary loop (MA–GCL). IgA nephropathy (IgAN) was the most frequent diagnosis (38.4 %), followed by membranous nephropathy (16.8 %) and focal segmental glomerulosclerosis (13.7 %). IgA deposition was universal in IgAN and IgA vasculitis nephritis (IgAVN), predominantly mesangial (84 % and 78 %, respectively), with MA–GCL co-deposition in 16–21 % of cases. Co-deposition with IgM (58 %), C3 (67 %), and C4d (15 %) was frequent in IgAN. Correlation analysis revealed significant associations between mesangial IgA intensity and mesangial IgM (ρ = 0.26), C3 (ρ = 0.61), C4d (ρ = 0.31), and glomerular C4d (ρ = 0.35) (all P < 0.05), while IgAVN showed additional correlation with fibronectin (ρ = 0.35). C4d positivity was also observed in hypertensive nephropathy (54.7 %). These findings define disease-specific IF profiles and highlight complement activation and fibronectin deposition as potential contributors to IgA-mediated glomerular injury. Integration of quantitative IF data with clinical outcomes may improve risk stratification and inform complement-targeted therapeutic strategies.

## Highlights

• IF staining profiles reveal varied immune deposit patterns in renal diseases.
• IgA deposition was hallmark in IgAN and IgAVN, with mesangial or co-deposition.
• Significant correlations found between IgA, IgM, C3, and C4d in IgAN mesangium.
• C3 and C4d deposition prevalent in glomerular diseases, with high C1q in LN.

## 1 Introduction

Histopathological evaluation of renal biopsy specimens remains the gold standard for diagnosing and managing renal parenchymal diseases, providing essential information on etiology, lesion characteristics, disease severity, prognosis, and potential therapeutic options [1]. Among histopathological techniques, immunofluorescence (IF) plays a pivotal role by enabling the detection and localization of a wide range of antigens within renal tissue. Correlation of IF findings with light microscopy, electron microscopy, and clinical data is critical for elucidating the nature and pathogenesis of renal lesions. For several glomerular diseases—including IgA nephropathy (IgAN), IgA vasculitis nephritis (IgAVN), C1q nephropathy, fibronectin glomerulopathy, and monoclonal immunoglobulin deposition disease—diagnosis depends almost entirely on immunomorphological findings [[2], [3], [4]].

IgA, the second most abundant serum immunoglobulin after IgG, plays a central role in mucosal immunity and contributes to autoimmune and inflammatory processes [5]. Genome-wide association studies have demonstrated shared genetic determinants between serum IgA levels and susceptibility to mucosal infections, celiac disease, inflammatory bowel disease, type 2 diabetes, IgAN, and other kidney diseases [6]. IgA-associated renal diseases are characterized by predominant or co-dominant IgA deposition—either as monomers, polymers, or immune complexes—in various glomerular compartments, most commonly the mesangium but also within capillary loops and along subepithelial, intramembranous, and subendothelial regions [[7], [8], [9], [10], [11]].

Despite its centrality to renal pathology, the immunofluorescence staining landscape remains incompletely characterized. Key knowledge gaps include the precise distribution patterns and staining intensities of IgA across different disease entities, the frequency and clinical significance of IgA co-deposition with other immunoglobulins or complement components, and the relationship between IF profiles and disease activity, prognosis, or response to therapy. Systematic characterizations of these IF patterns are needed to refine diagnostic accuracy, clarify pathogenic mechanisms, and inform personalized treatment strategies in glomerular diseases.

## 2 Materials and methods

This retrospective study reviewed renal biopsy records from a single tertiary medical center in Changsha, China, spanning August 2011 to November 2022. The study was approved by the Medical Ethics Committee of the Second Xiangya Hospital, Central South University (Project ID: LYF2022111, approved 07-05-2023) and conducted in accordance with the Declaration of Helsinki. Written informed consent was obtained from all participants prior to data collection. Clinical and histopathological data were systematically extracted for each case. Among 12,102 consecutive biopsies performed during this period, cases were excluded if patients were younger than 14 years, had repeat biopsies, contained fewer than 10 glomeruli, had incomplete demographic or immunofluorescence data, or were diagnosed with non-glomerular/unclassified lesions (including minor glomerular abnormalities). After these exclusions, 10,489 biopsies were included in the final analysis.

All specimens underwent standardized evaluation using light microscopy and immunofluorescence microscopy, with electron microscopy performed in approximately 90 % of cases. Immunofluorescence on frozen tissue (IF–F) was conducted using antibodies against immunoglobulin heavy and light chains (IgA, IgG, IgM, kappa, and lambda), complement pathway components (C1q, C3, and C4d), and fibrinogen (Fib). The intensity of deposits was semi-quantitatively graded on a five-point scale: − (negative), 1+ (weak), 2+ (moderate), 3+ (strong), and 4+ (very strong). Assessments were performed by a histopathologist blinded to clinical data and independently reviewed by two experienced nephropathologists. Final diagnoses were established by consensus during multidisciplinary pathology conferences involving nephrologists and nephropathologists.

Statistical analyses were performed using IBM SPSS Statistics 25 (Armonk, NY: IBM Corp.). Non-parametric correlation analyses were conducted using Spearman's rho and Kendall's tau, chosen because the data were ordinal or not normally distributed. Spearman's rho evaluates monotonic associations between ranked variables, while Kendall's tau is more robust to tied ranks and provides a complementary measure of correlation. A two-sided p-value <0.05 was considered statistically significant.

## 3 Results

### 3.1 Demographic characteristics of patients

**Fig. 1:**
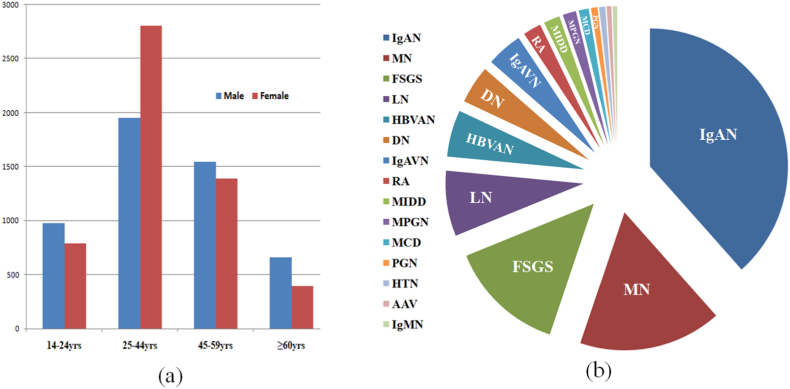
(**a**) The distribution of renal biopsy samples by age group and gender, (**b**)The frequencies of biopsy-proven glomerular diseases in 2011–2022. IgAN: IgA nephropathy, IgAVN: IgA vasculitis nephritis, MIDD:Monoclonal immunoglobulin deposition disease, LN:Lupus nephritis, HBVAN:HBV-associated nephropathy, DN:Diabetic Nephropthy, EPGN:Endocapillary proliferative glomerulonephritis, RA:Renal amyloidosis, MN: Membranous nephropathy, MPGN: Membranoproliferative glomerulonephritis, HTN: Hypertensive nephropathy without IgAN, MCD: Minimal change disease, IgMN: IgM nephropathy, AGN:ANCA-associated glomerulonephritis or **ANCA**-associated vasculitis with renal involvement, FSGS: Focal segmental glomerulosclerosis.

A total of 10,489 consecutive native kidney biopsy records were included in the final analysis. The overall male-to-female ratio was 1:1.07. The mean age at biopsy was 39.1 ± 14.6 years (range: 14–83 years). Patients were stratified into four age categories: 14–24, 25–44, 45–59, and ≥60 years. The distribution of biopsy samples by age group and sex, along with the frequencies of biopsy-proven glomerular diseases, is presented in Fig. 1. The highest number of biopsies was performed in the 25–44-year age group, with a male-to-female ratio of 1:1.44 in this cohort.

Overall, IgAN was the most frequently diagnosed biopsy-proven glomerular disease, accounting for 38.43 % of cases, followed by membranous nephropathy (MN, 16.76 %) and focal segmental glomerulosclerosis (FSGS, 13.65 %). Table 1 summarizes the demographic characteristics of patients undergoing biopsy, categorized by disease type and age group.

**Table 1:** Demographic characteristics of patients who underwent kidney biopsy procedures.

Renal disease^a^	Age group	14–24		25–44		45–59		≥60		M/F ratio
	N of cases	Male	Female	Male	Female	Male	Female	Male	Female	
IgAN	4031	382(9.48 %)	377(9.35 %)	927(23.00 %)	1454(36.07 %)	328(8.14 %)	434(10.77 %)	74(1.84 %)	55(1.36 %)	0.74
IgAVN	465	120(25.81 %)	97(20.86 %)	59(12.69 %)	87(18.71 %)	23(4.95 %)	42(8.03 %)	23(4.95 %)	14(3.01 %)	0.94
MIDD	200	11(5.50 %)	7(3.50 %)	37(18.50 %)	60(30 %)	27(13.5 %)	24(12.00 %)	19(9.50 %)	15(7.50 %)	0.89
LN	803	103(12.83 %)	109(13.57 %)	52(6.48 %)	382(47.57 %)	22(2.74 %)	117(14.57 %)	2(0.25 %)	16(1.99 %)	0.26
HBVAN	575	18(3.13 %)	18(3.13 %)	159(27.65 %)	98(17.04 %)	132(22.96 %)	74(12.87 %)	51(8.87 %)	25(4.35 %)	1.67
DN	466	2(0.43 %)	3(0.64 %)	85(18.24 %)	29(6.22 %)	174(37.34 %)	75(16.09 %)	61(13.09 %)	37(7.94 %)	2.24
EPGN	80	33(41.25 %)	21(26.25 %)	4(5.00 %)	12(15.00 %)	2(2.50 %)	3(3.75 %)	4(5.00 %)	1(1.25 %)	1.16
RA	223	6(2.69 %)	1(0.45 %)	17(7.62 %)	34(15.25 %)	45(20.18 %)	32(14.35 %)	55(24.66 %)	33(14.80 %)	1.23
MN	1758	46(2.62 %)	44(2.50 %)	287(16.33 %)	200(11.38 %)	518(29.47 %)	303(17.24 %)	225(12.80 %)	135(7.68 %)	1.58
MPGN	164	29(17.68 %)	15(9.15 %)	23(14.02 %)	52(31.71 %)	13(7.93 %)	23(14.02 %)	6(3.66 %)	3(1.83 %)	0.76
HTN	75	0(0.00 %)	0(0.00 %)	20(26.67 %)	7(9.33 %)	24(32.00 %)	7(9.33 %)	13(7.33 %)	4(5.33 %)	3.17
MCD	124	43(34.68 %)	11(8.87 %)	18(14.52 %)	27(21.77 %)	7(5.65 %)	10(8.06 %)	5(4.03 %)	3(2.42 %)	1.43
IgMN	56	10(17.86 %)	7(12.50 %)	7(12.50 %)	15(26.79 %)	4(7.14 %)	6(10.71 %)	4(7.14 %)	3(5.36 %)	0.81
AGN	58	0(0 %)	1(1.72 %)	4(6.90 %)	10(17.24 %)	17(29.31 %)	12(20.69 %)	7(12.07 %)	7(12.07 %)	0.93
FSGS	1432	172(12.01 %)	83(5.80 %)	253(17.67 %)	333(23.25 %)	206(14.39 %)	226(15.78 %)	113(7.89 %)	46(3.21 %)	1.08

a IgAN: IgA nephropathy, IgAVN: IgA vasculitis nephritis, MIDD:Monoclonal immunoglobulin deposition disease, LN:Lupus nephritis, HBVAN:HBV-associated nephropathy, DN:Diabetic Nephropthy, EPGN:Endocapillary proliferative glomerulonephritis, RA:Renal amyloidosis, MN: Membranous nephropathy, MPGN: Membranoproliferative glomerulonephritis, HTN: Hypertensive nephropathy without IgAN, MCD: Minimal change disease, IgMN: IgM nephropathy, AGN:ANCA-associated glomerulonephritis or **ANCA**-associated vasculitis with renal involvement, FSGS: Focal segmental glomerulosclerosis.

Hypertensive nephropathy was more commonly observed in men. Similarly, diabetic nephropathy (DN), HBV-associated nephropathy (HBVAN), MN, and minimal change disease (MCD) were more prevalent in male patients, whereas lupus nephritis (LN) was significantly more frequent among women. Endocapillary proliferative glomerulonephritis (EPGN), IgA vasculitis nephritis (IgAVN), and MCD occurred predominantly in young adults. IgAN, FSGS, monoclonal immunoglobulin deposition disease (MIDD), membranoproliferative glomerulonephritis (MPGN), and immunoglobulin M nephropathy (IgMN) were most common in the 25–44-year age group. LN was more prevalent in both adolescent and adult women, while the prevalence of renal amyloidosis (RA) increased progressively with age.

### 3.2 Immunoglobulins and complement components deposition and distribution in glomerular diseases

Based on the reported localization of immune deposits within the glomeruli, we categorized the IF staining patterns of immunoglobulin and complement component deposition into three groups: mesangial area only (MAO), glomerular capillary loops only (GCLO), and combined mesangial-capillary loop co-staining (MA–GCL). The frequency and distribution of IgA deposition varied considerably across different renal diseases (Table 2).

**Table 2:** Distribution and frequency of IgA deposition across major renal diseases.

Glomerular disease	Total cases	N^a^ of IgA deposits	IgA Deposits rate	MAO		GCLO		MA-GCL	
				N	%	N	%	N	%
IgAN	4031	4031	1.00	3386	84.00	0	0.00	645	16.00
IgAVN	444	444	1.00	345	77.70	7	1.58	92	20.72
MIDD	200	159	0.80	120	75.47	11	6.92	28	17.61
LN	803	602	0.75	81	13.46	221	36.71	300	49.83
HBVAN	575	385	0.67	201	52.21	94	24.42	90	23.38
DN	466	239	0.51	61	25.52	97	40.59	81	33.89
EPGN	80	39	0.49	7	17.95	18	46.15	14	35.90
RA	223	96	0.43	69	71.88	8	8.33	19	19.79
MN	1758	602	0.34	76	12.62	396	65.78	130	21.59
MPGN	164	55	0.34	24	43.64	8	14.55	23	41.82
HTN	75	22	0.29	8	36.36	8	36.36	6	27.27
MCD	124	34	0.27	20	58.82	4	11.76	10	29.41
IgMN	56	14	0.25	8	57.14	0	0.00	6	42.86
AGN	58	14	0.24	3	21.43	3	21.43	8	57.14
FSGS	1432	302	0.21	120	39.74	61	20.20	121	40.07

a N: Number, MAO: mesangial area only, GCLO: glomerular capillary loops only, MA-GCL: co-staining in mesangial area and glomerular capillary loops. %: percentage.

As expected, IgA deposition was universally present in IgAN and IgAVN, collectively referred to as “IgA-associated renal diseases.” Elevated IgA deposition rates were also observed in several other conditions, including MIDD (80 %), LN (75 %), HBVAN (67 %), DN (51 %), and EPGN (49 %).

The anatomical distribution of IgA deposits displayed distinct disease-specific patterns. In IgAN, mesangial IgA deposition is the hallmark histopathological feature, with approximately 16 % of cases also demonstrating involvement of the GCL. IgAVN exhibited a slightly higher frequency of MA–GCL co-deposition (20.72 %), suggesting a broader distribution of immune deposits.

Outside of IgAN and IgAVN, mesangial-predominant IgA deposition was most commonly observed in MIDD, HBVAN, RA, and IgMN, whereas GCL-predominant IgA deposition was characteristic of LN, DN, EPGN, and MN.

In addition to IgA, the distribution of other immunoglobulin deposits, as assessed by the standard IF protocol, is summarized in Table 3. MN (97 %) and LN (87 %) demonstrated predominant IgG deposition, with most deposits localized to the GCL region. Other diseases, including EPGN (68 %), DN (65 %), and HBVAN (64 %), also exhibited IgG deposition in the GCL region.

**Table 3:** Distribution of IgG, IgM, and fibronectin deposits in glomerular diseases.

Glomerular disease	Total cases	N^a^ of IgG deposits	IgG Deposits rate	MAO		GCLO		MA-GCL		IgM deposition		Fib	
				N	%	N	%	N	%	N	%	N	%
IgAN	4031	1191	0.30	715	60.03	115	9.66	361	30.31	2351	58.32	992	24.61
IgAVN	444	137	0.31	71	51.82	25	18.25	41	29.93	256	57.66	201	45.27
MIDD	200	85	0.43	23	27.06	39	45.88	23	27.06	122	61.00	63	31.50
LN	803	701	0.87	34	4.85	338	48.22	329	46.93	555	69.12	212	26.40
HBVAN	575	366	0.64	46	12.57	249	68.03	71	19.40	339	58.96	138	24.00
DN	466	301	0.65	13	4.32	236	78.41	52	17.28	232	49.79	81	17.38
EPGN	80	54	0.68	5	9.26	30	55.56	19	35.19	30	37.50	15	18.75
RA	223	74	0.33	32	43.24	22	29.73	20	27.03	105	47.09	27	12.11
MN	1758	1700	0.97	12	0.71	1547	91.00	141	8.29	732	41.64	330	18.77
MPGN	164	56	0.34	11	19.64	19	33.93	26	46.43	64	39.02	13	7.93
HTN	75	27	0.36	3	11.11	18	66.67	6	22.22	35	46.67	11	14.67
MCD	124	17	0.14	2	11.76	10	58.82	5	29.41	50	40.32	6	4.84
IgMN	56	17	0.30	1	5.88	7	41.18	9	52.94	56	100	1	1.79
AGN	58	20	0.34	1	5.00	8	40.00	11	55.00	23	39.66	5	8.62
FSGS	1432	391	0.27	45	11.51	237	60.61	109	27.88	535	37.36	91	6.35

a N: Number, MAO: mesangial area only, GCLO: glomerular capillary loops only, MA-GCL: co-staining in mesangial area and glomerular capillary loops.%: percentage.

By contrast, IgAN, IgAVN, and RA **demonstrated** lower frequencies of IgG deposition, with a preference for mesangial localization. Compared with IgAN, IgAVN exhibited a higher frequency of fibronectin deposits. With the exception of IgMN, IgM deposition demonstrated heterogeneous distribution patterns across different glomerular diseases.

Complement component deposition across glomerular diseases is summarized in Table 4. EPGN (90 %) exhibited the highest C3 deposition rate, primarily localized to the GCL or MA–GCL regions. LN (76 %), HBVAN (67 %), and MN (58 %) also **demonstrated** C3 deposition with a similar distribution pattern. In contrast, C3 deposition in IgAN (68 %), MIDD (72 %), IgAVN (61 %), and RA (32 %) was predominantly restricted to the mesangial region. A high frequency of C1q deposition was characteristic of LN. Interestingly, HTN demonstrated a notably elevated C4d deposition rate (54.67 %).

**Table 4:** Distribution of complement component deposits across glomerular diseases.

Glomerular disease	Total cases	N^a^ of C3 deposits	C3 Deposits rate	MAO		GCLO		MA-GCL		C1q deposition		C4d deposition	
				N	%	N	%	N	%	N	%	N	%
IgAN	4031	2731	0.68	2270	83.12	31	1.14	430	15.75	725	17.99	611	15.16
IgAVN	444	271	0.61	204	75.28	11	4.06	56	20.66	73	16.44	57	12.84
MIDD	200	144	0.72	92	63.89	21	14.58	31	21.53	67	33.50	59	29.50
LN	803	614	0.76	155	25.24	206	33.55	253	41.21	662	82.44	79	9.84
HBVAN	575	387	0.67	126	32.56	170	43.93	910	23.51	266	46.26	69	12.00
DN	466	204	0.44	35	17.16	103	50.49	66	32.35	169	36.27	104	22.32
EPGN	80	72	0.90	10	13.89	30	41.67	32	44.44	24	30.00	6	7.50
RA	223	71	0.32	43	60.56	12	16.90	16	22.54	75	33.63	64	28.70
MN	1758	1015	0.58	16	1.58	951	93.69	48	4.73	900	51.19	220	12.51
MPGN	164	29	0.18	8	27.59	5	17.24	16	55.17	22	13.41	16	9.76
HTN	75	26	0.35	6	36.36	11	36.36	9	27.27	26	34.67	41	54.67
MCD	124	12	0.10	5	41.67	5	41.67	2	16.67	19	15.32	17	13.71
IgMN	56	5	0.09	1	20.00	0	0.00	4	80.00	11	19.64	0	0
AGN	58	24	0.41	2	8.33	4	16.67	18	75.00	16	27.59	3	5.17
FSGS	1432	212	0.15	75	35.38	62	29.25	75	35.38	198	13.83	201	14.04

a N: Number, MAO: mesangial area only, GCLO: glomerular capillary loops only, MA-GCL: co-staining in mesangial area and glomerular capillary loops.%.

### 3.3 IgA co-deposits and their correlation with IgG, IgM, C3, C1q, C4d, and Fib in IgA-associated renal diseases

IgA deposition is a hallmark of IgA-associated renal disorders, including IgAN and IgAVN (Table 5). Approximately one-quarter of cases in both conditions **demonstrated** co-deposition with IgG in the mesangial region, but IgAN exhibited a higher proportion of GCL co-deposits compared with IgAVN (73.8 % vs. 39.39 %). Co-deposition patterns with IgM, C1q, C3, and C4d were similar in both diseases, whereas IgAVN **demonstrated** more frequent fibronectin co-deposition.

**Table 5:** Co-deposition of IgA with other immune components in IgA-Associated renal diseases.

Disease	Location of IgA deposition	IgG		IgM		C1q		C3		C4d		Fib	
		N	%	N	%	N	%	N	%	N	%	N	%
IgAN	MA (4031)	1076	26.69	2351	58.32	725	17.99	2700	66.98	611	15.16	992	24.61
	GCL(645)	476	73.80	382	59.22	158	24.50	254	39.38	3	0.47	162	25.12
IgAVN	MA(437)	109	24.94	252	57.67	71	16.25	257	58.81	56	12.81	200	**45.77**
	GCL(99)	39	39.39	69	69.70	23	23.23	46	46.46	2	2.02	41	41.41

**Fig. 2:**
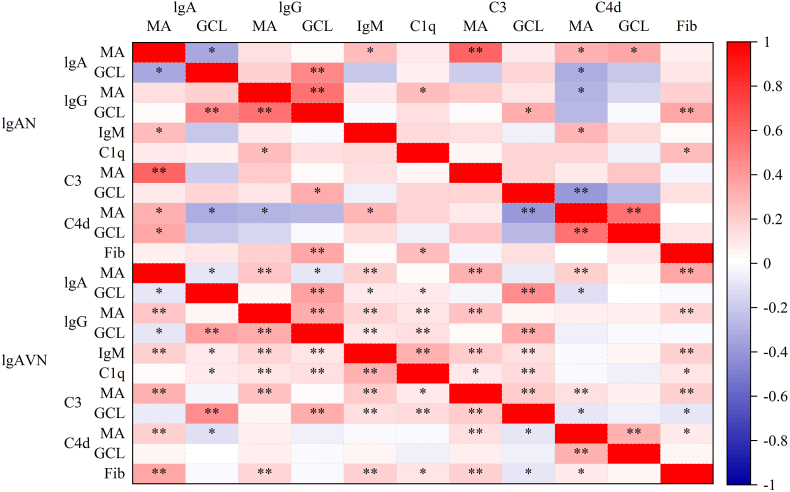
Heatmap of correlations between IgA deposits and other immune components in IgA-associated renal diseases.

Non-parametric correlation analysis, visualized as a heatmap (Fig. 2), revealed disease-specific associations between IgA deposits and other immune components, highlighting patterns of co-deposition and potential interactions among immunoglobulins, complement components, and fibronectin in IgA-associated renal diseases. In IgAN, mesangial IgA intensity correlated positively with mesangial IgM (r = 0.260, P < 0.05), C3 (r = 0.614, P < 0.01), and C4d (r = 0.308, P < 0.05), as well as GCL C4d (r = 0.345, P < 0.05). In IgAVN, mesangial IgA intensity correlated with mesangial IgG (r = 0.225, P < 0.01), IgM (r = 0.198, P < 0.01), C3 (r = 0.315, P < 0.01), C4d (r = 0.181, P < 0.01), and fibronectin (r = 0.354, P < 0.01). Additionally, in IgAN, GCL IgG and C1q deposition were associated with fibronectin.

The heatmap illustrates non-parametric correlation coefficients (r values) between IgA deposit intensity and the intensity of IgG, IgM, C1q, C3, C4d, and fibronectin in the mesangial area (MA) and glomerular capillary loops (GCL) in IgAN and IgAVN. Positive correlations are indicated by warmer colors, while negative correlations are indicated by cooler colors. Statistically significant correlations are highlighted (∗P < 0.05, ∗∗P < 0.01). This visualization highlights patterns of co-deposition and potential interactions among immune components in IgA-associated renal disorders.

## 4 Discussion

Renal biopsy remains the gold standard for diagnosing, staging, and prognosticating renal parenchymal diseases. In this retrospective study, we analyzed IF staining profiles across a spectrum of renal disorders using the Renal Biopsy Registry of the Department of Nephrology at Second Xiangya Hospital (2011–2022). IgAN was the most prevalent glomerular disease (38.43 %), followed by MN, (16.76 %) and FSGS (13.65 %). By contrast, Brazilian cohorts report FSGS as the most common (37.3 %), followed by IgAN (24.4 %) and MN (18.6 %) [12]. Over the past decade in China, IgAN has remained the most frequent glomerular disorder, with a marked increase in MN cases [[13], [14], [15]].

Patient demographics revealed distinct age- and sex-specific patterns. HTN, DN, HBVAN, MN, and MCD were more prevalent in men, whereas LN predominated in women. Younger adults were more likely to present with EPGN, IgAVN, and MCD, while IgAN, FSGS, MIDD, MPGN, and IgMN predominated in the 25–44-year age group. RA prevalence increased with age. Despite geographic variation in sex ratios, no significant gender differences were observed in this cohort [16,17].

Immune deposition patterns were classified by localization as MAO, GCLO, or MA–GCL. IgA deposition varied by disease: it was universal in IgAN and IgAVN, and frequent in MIDD (80 %), LN (75 %), HBVAN (67 %), DN (51 %), and EPGN (49 %). IgAN predominantly **demonstrated** mesangial IgA deposits, whereas IgAVN exhibited more MA–GCL co-deposition. MIDD, HBVAN, RA, and IgMN were mainly mesangial, while LN, DN, EPGN, and MN **demonstrated** predominantly GCL deposits. Latent mesangial IgA deposition in the absence of clinical nephropathy has been reported in 3–24.5 % of healthy allograft donors and unselected autopsy series. Given this high background prevalence, the detection of IgA deposits alone is neither diagnostic nor prognostic. Interpretation should instead rely on the distribution pattern, staining intensity, and co-deposition of complement or other immunoglobulins to better determine pathogenic relevance and distinguish incidental findings from clinically significant disease [[18], [19], [20], [21]].

Other immunoglobulin and complement deposits varied across diseases. MN and LN **demonstrated** predominant IgG in GCL, whereas IgG in IgAN, IgAVN, and RA was mainly mesangial. GCL IgG deposits in IgAN have been associated with poor prognosis [22]. Fibronectin was more frequent in IgAVN, while IgM deposition varied across diseases. C3 deposition was common, with EPGN **demonstrating** the highest rate, primarily in GCL or MA–GCL. LN, HBVAN, and MN followed similar patterns, while IgAN, MIDD, IgAVN, and RA were predominantly mesangial. High C1q deposition characterized LN, and HTN unexpectedly exhibited elevated C4d (54.67 %). Complement dysregulation contributes to inflammation and disease progression, as observed in atypical hemolytic uremic syndrome (aHUS), C3 glomerulopathy (C3GN), DN, and MN [23,24].

In IgA-associated diseases, co-deposition of IgA with IgG, IgM, C1q, C3, C4d, and fibronectin revealed disease-specific patterns. Approximately one-quarter of IgAN and IgAVN cases had mesangial IgG co-deposition, with IgAN **demonstrating** higher GCL co-deposits (73.8 % vs. 39.4 %). In IgAN, mesangial IgA intensity correlated with mesangial IgM, C3, C4d, and GCL C4d, whereas IgAVN **demonstrated** correlations with mesangial IgG, IgM, C3, C4d, and fibronectin. GCL IgG and C1q deposition in IgAN also correlated with fibronectin. Notably, GCL localization is linked to increased risk of end-stage renal disease (ESRD), transplantation, death, or doubling of serum creatinine [25].

Zagorec et al. reported that 45.3 % of primary IgAN patients had glomerular C4d deposits, which independently predicted kidney failure (HR 5.87, p = 0.032). C4d-positive patients exhibited higher blood pressure, proteinuria, and serum urate, with lower eGFR and worse renal survival [26]. These findings support C4d as a marker of disease severity and a potential target for complement-directed therapies. Combined with other complement markers, C4d may help stratify prognosis and elucidate distinct complement activation pathways in glomerular diseases [27].

Higher deposition of IgA, IgG, C3, and fibronectin correlates with more severe glomerular injury, including crescents and mesangial/endocapillary hypercellularity. IgM deposition is associated with severe pathology and adverse outcomes, including lower serum albumin and eGFR, and higher cholesterol, proteinuria, and serum IgM [28,29]. Recent cohort data indicate that ∼70 % of patients with advanced IgAN had mesangial IgM deposits, with significantly higher progression to *End-stage kidney disease* (ESKD). Multivariate analysis identified low eGFR, higher Oxford MEST-C scores, and mesangial IgM deposition as independent prognostic factors [30].

## 5 Limitations

This study benefits from a large, single-center cohort with standardized IF assessment, providing detailed insights into immune deposition patterns. Nevertheless, several limitations should be acknowledged: the retrospective, single-center design and inclusion of only biopsy-proven cases introduce potential selection bias; the semi-quantitative nature of IF scoring and inter-observer variability may affect reproducibility; and the absence of systematic longitudinal follow-up and mechanistic investigations precludes definitive conclusions regarding the prognostic and causal significance of immune deposits.

## 6 Conclusions

This study elucidates the prevalence and IF profiles of glomerular diseases, revealing distinct immunoglobulin and complement deposition patterns. These findings underscore the critical role of IF in diagnosis and prognosis, particularly in IgA-associated disorders, where biomarkers like C4d and IgM **demonstrate** promise for risk stratification. The results provide valuable insights into disease mechanisms and progression. Future multicenter, prospective studies with quantitative IF analyses and mechanistic investigations are essential to clarify causal pathways, validate biomarkers, and inform personalized therapeutic strategies.

## Informed consent statement

Informed consent was obtained from all subjects involved in the study.

## Institutional review board statement

The study was conducted in accordance with the Declaration of Helsinki, and approved by the Medical Ethics Committee at the Second Xiangya Hospital of Central South University (Project identification number: LYF2022111 on [07-05-2023]).

## Funding

This research was supported by grant (02250041) from China Guanghua Foundation.

## CRediT authorship contribution statement

**Jiarong Song:** Data curation, Validation, Writing – original draft. **Xinyuan Cui:** Data curation, Validation. **Shuguang Yuan:** Resources. **Hong Liu:** Resources. **Yu Liu:** Resources. **Xuan Zhou:** Formal analysis. **Lin Sun:** Resources. **Xuejing Zhu:** Conceptualization, Validation, Writing – review & editing. **Yifu Li:** Conceptualization, Funding acquisition, Supervision, Writing – review & editing.

## Declaration of competing interest

The authors declare the following financial interests/personal relationships which may be considered as potential competing interests:Yifu Li reports financial support was provided by China Guanghua Technology Foundation. If there are other authors, they declare that they have no known competing financial interests or personal relationships that could have appeared to influence the work reported in this paper.

## Figures and Tables

**Table:** 

IgAN	IgA nephropathy
IgAVN	IgA vasculitis nephritis
MIDD	Monoclonal immunoglobulin deposition disease
LN	Lupus nephritis,
HBVAN	HBV-associated nephropathy
DN	Diabetic Nephropthy
EPGN	Endocapillary proliferative glomerulonephritis
RA	Renal amyloidosis
MN	Membranous nephropathy
MPGN	Membranoproliferative glomerulonephritis
HTN	Hypertensive nephropathy without IgAN
MCD	Minimal change disease
IgMN	IgM nephropathy
AGN	ANCA-associated glomerulonephritis or **ANCA**-associated vasculitis with renal involvement
FSGS	Focal segmental glomerulosclerosis
MAO	mesangial area only
GCLO	glomerular capillary loops only
MA-GCL	co-staining in mesangial area and glomerular capillary loops
	

## Data Availability

Data supporting this study are available from the corresponding author on approval and a data sharing agreement.
